# EDIL3 promotes epithelial–mesenchymal transition and paclitaxel resistance through its interaction with integrin α_V_β_3_ in cancer cells

**DOI:** 10.1038/s41420-020-00322-x

**Published:** 2020-09-16

**Authors:** J. Gasca, M. L. Flores, R. Jiménez-Guerrero, M. E. Sáez, I. Barragán, M. Ruíz-Borrego, M. Tortolero, F. Romero, C. Sáez, M. A. Japón

**Affiliations:** 1 Instituto de Biomedicina de Sevilla (IBIS), Hospital Universitario Virgen del Rocío/CSIC/Universidad de Sevilla, 41013 Seville, Spain; 2 Centro Andaluz de Estudios Bioinformáticos (CAEBi), 41013 Seville, Spain; 3 Department of Physiology and Pharmacology, Karolinska Institutet, 17177 Stockholm, Sweden; 4 Section of Immuno-Oncology, Medical Oncology, Hospitales Universitarios Regional y Virgen de la Victoria, Instituto de Investigación Biomédica de Málaga (IBIMA), 29010 Malaga, Spain; 5 Department of Medical Oncology, Hospital Universitario Virgen del Rocío, 41013 Seville, Spain; 6 Department of Microbiology, Faculty of Biology, Universidad de Sevilla, 41012 Seville, Spain; 7 Department of Pathology, Hospital Universitario Virgen del Rocío, 41013 Seville, Spain

**Keywords:** Prognostic markers, Oncogenesis, Translational research

## Abstract

Epithelial–mesenchymal transition (EMT) has recently been associated with tumor progression, metastasis, and chemotherapy resistance in several tumor types. We performed a differential gene expression analysis comparing paclitaxel-resistant vs. paclitaxel-sensitive breast cancer cells that showed the upregulation of *EDIL3* (EGF Like Repeats and Discoidin I Like Domains Protein 3). This gene codifies an extracellular matrix protein that has been identified as a novel regulator of EMT, so we studied its role in tumor progression and paclitaxel response. Our results demonstrated that EDIL3 expression levels were increased in paclitaxel-resistant breast and prostate cancer cells, and in subsets of high-grade breast and prostate tumors. Moreover, we observed that EDIL3 modulated the expression of EMT markers and this was impaired by cilengitide, which blocks the EDIL3–integrin α_V_β_3_ interaction. EDIL3 knockdown reverted EMT and sensitized cells to paclitaxel. In contrast, EDIL3 overexpression or the culture of cells in the presence of EDIL3-enriched medium induced EMT and paclitaxel resistance. Adding cilengitide resensitized these cells to paclitaxel treatment. In summary, EDIL3 may contribute to EMT and paclitaxel resistance through autocrine or paracrine signaling in cancer cells. Blockade of EDIL3–integrin α_V_β_3_ interaction by cilengitide restores sensitivity to paclitaxel and reverts EMT in paclitaxel-resistant cancer cells. Combinations of cilengitide and taxanes could be beneficial in the treatment of subsets of breast and prostate cancers.

## Background

Epithelial–mesenchymal transition (EMT) is the process by which epithelial cells loose the adherent and tight junctions that keep them in contact with their neighbor cells to gain a mesenchymal phenotype. This transition favors an increased mobility, migration, or invasion. EMT is a complex process involving several transcription factors, cell-surface and cytoskeletal proteins, components of the extracellular matrix (ECM), and numerous signaling pathways, including Wnt/β-catenin, TGF-β, Hedgehog, and integrin pathways^1,2^. Among the transcriptional factors involved, members of the Snail family (SNAI1, SNAI2/Slug, and Twist) are considered to be key regulators of EMT. Concretely, SNAI1 directly represses E-cadherin expression that provides the physical structure for both cell–cell junctions and for the recruitment of signaling complexes in epithelial cells^2,3^. Thus, the loss of E-cadherin expression is one of the earliest events in the EMT process and, together with the gain of vimentin expression, is commonly used to demonstrate EMT in experimental situations^1,4^. Although EMT is a key process during embryogenesis, wound healing and tissue regeneration, it has recently been suggested to play an important role in tumor progression and metastasis^1,2,4^. In breast cancer, EMT is associated with unfavorable prognosis and metastasis, and it has been proposed that EMT can be responsible for the spreading of breast epithelial tumor cells to the liver, lungs, or bone marrow^5,6^. Moreover, resistance to paclitaxel, docetaxel, doxorubicin, and tamoxifen has been linked with the aberrant expression of the EMT-related genes Twist, Snail, and Slug, as well as with the induction of EMT in breast cancer cells^7–10^. Finally, EMT has been associated with the aggressive behavior and resistance to docetaxel therapy in advanced prostate cancer^11,12^.

EGF Like Repeats and Discoidin I Like Domains Protein 3 (EDIL3), also known as Developmental Endothelial Locus 1, is an ECM protein containing three EGF-like domains; the second one has a RGD motif (Arg–Gly–Asp) that allows the interaction of EDIL3 with integrins. EDIL3 acts as a pro-angiogenic factor, mediator of the immune and anti-inflammatory response, and a regulator of endothelial cell adhesion and migration^13–15^. It has been reported that EDIL3 is overexpressed in several tumor types, including bladder, pancreas, breast, and liver carcinomas, and associates with tumor progression and poor prognosis^16–23^. EDIL3 has been identified as a novel regulator of EMT in hepatocellular carcinoma cells, as the increased expression of EDIL3 by miRNA-137 downregulation triggers ERK and TGF-β activation via interaction with the integrin α_V_β_3_ (^20^). This integrin plays a crucial role in the growth of brain metastasis in breast cancer^24^. Also, EDIL3 has been identified in the extracellular vesicles of breast cancer cells that may be used for early breast cancer detection in the plasma of patients^21–25^.

Since EDIL3 is a regulator of EMT and both EDIL3 and EMT play important roles in the progression of cancer and acquisition of resistance to chemotherapy, we studied the expression of EDIL3 and its impact on paclitaxel resistance. Given that the use of paclitaxel is restricted by acquisition of resistance^26^ and that pharmacological agents that inhibit the activation of integrins, such as cilengitide, are available, we also examined if the combination of both agents improved the apoptotic response of breast and prostate cancer cells with the aim of designing new therapeutic strategies for the treatment of cancer.

## Results

### EMT-related genes are differentially expressed in paclitaxel-sensitive MDA-MB-468 vs. paclitaxel-resistant MDA-MB-468R breast cancer cells

**Fig. 1:**
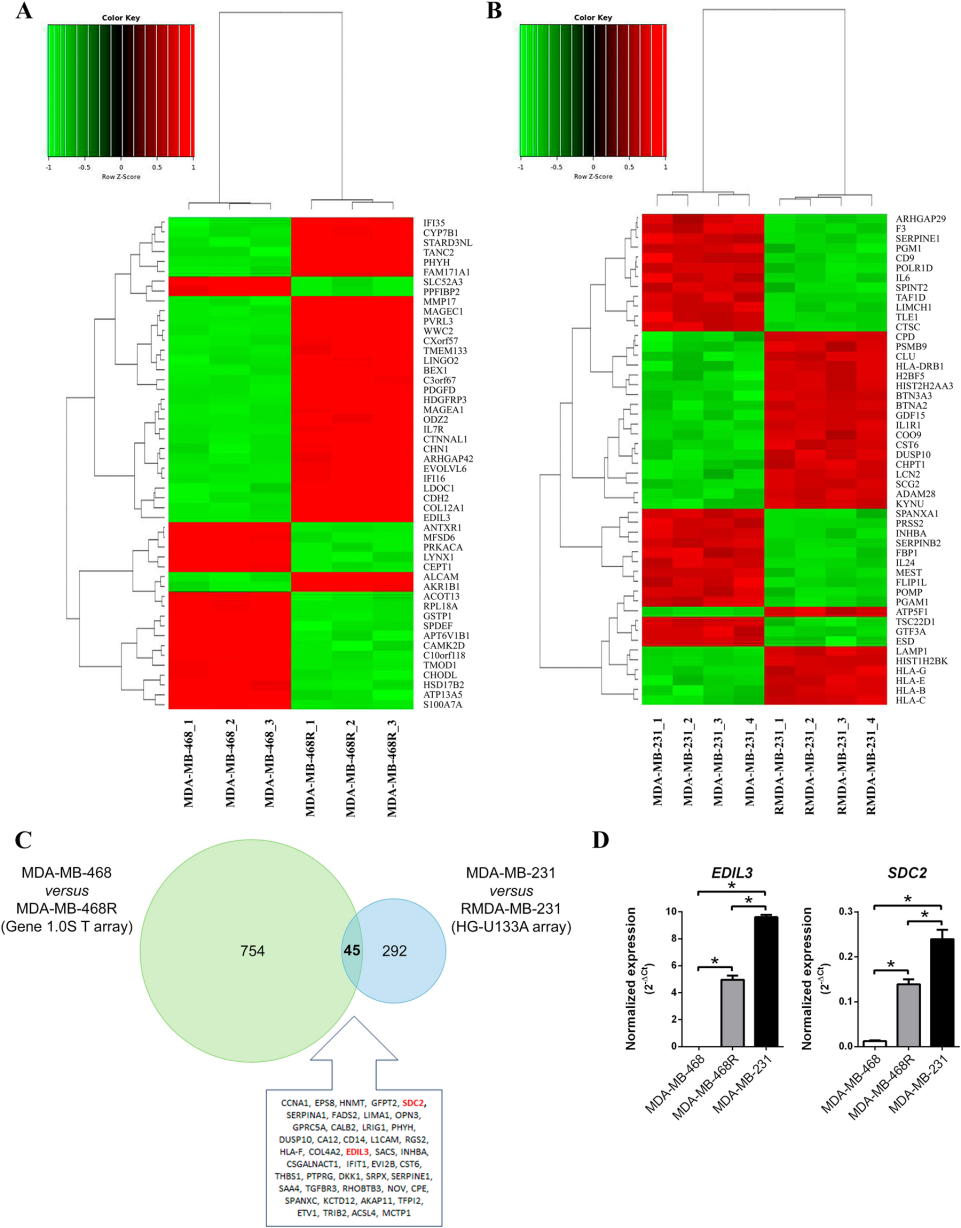
Epithelial–mesenchymal transition-related genes are differentially expressed in paclitaxel-sensitive vs. paclitaxel-resistant breast cancer cells. **a** Heat-map log2 representing the top 50 differentially expressed genes in paclitaxel-sensitive MDA-MB-468 vs. paclitaxel-resistant MDA-MB-468R cells. Each column represents a sample and each row represents a gene. Red are upregulated genes and green are downregulated genes. **b** Heat-map log2 representing the top 50 differentially expressed genes in MDA-MB-231 and paclitaxel-resistant RMDA-MB-231 cells obtained from the dataset E-GEPD-1279. Each column represents a sample and each row represents a gene. Red are upregulated genes and green are downregulated genes. **c** Venn diagram representing differentially expressed genes from MDA-MB-468 vs. MDA-MB-468R comparison in green, and from MDA-MB-231 vs. RMDA-MB-231 comparison in blue. List of 45 common differentially expressed genes is presented. **d** qRT-PCR analysis of *EDIL3* and *SDC2* genes in MDA-MB-468, MDA-MB-468R, and MDA-MB-231 breast cancer cells. The quantity of each transcript was divided by the quantity of *TBP* and *EIF2B2* to obtain a normalized value. Data are presented as mean ± SEM (*n* ≥ 3). *P < 0.05 from Student’s *t* test; NS not significant.

The whole-genome expression profiles of paclitaxel-sensitive MDA-MB-468 and paclitaxel-resistant MDA-MB-468R, were analyzed with the aim of identifying genes that are involved in the acquisition of paclitaxel resistance. Affymetrix Human Gene 1.0 ST Array analyses of mRNA isolated from MDA-MB-468 and MDA-MB-468R cells allowed the identification of 799 genes with differential expression. Out of these genes, 353 were downregulated, whereas 446 were upregulated in MDA-MB-468R vs. MDA-MB-468, including *EDIL3*, *SNAI2*/*Slug*, *SPOCK1*, and *Vimentin*, all of which are involved in EMT (Fig. 1a and Supplementary Table S1).

The available dataset E-GEOD-12791 contains the whole-genome expression profiles of MDA-MB-231 breast cancer cell line, and its derived paclitaxel-resistant RMDA-MB-231 cell line analyzed with the Affymetrix Human Genome U133A Array. In this dataset, out of 337 differentially expressed genes, 159 were downregulated and 178 were upregulated in RMDA-MB-231 vs. MDA-MB-231 (Fig. 1b and Supplementary Table S2). *EDIL3* and *SDC2* were among the upregulated genes in both paclitaxel-resistant MDA-MB-468R and RMDA-MB-231 cells, showing a strong significance in the meta-analysis of both datasets (adjusted *P* < 10^−15^; Fig. 1c and Supplementary Table S3).

We validated *EDIL3* and *SDC2* gene expression by quantitative real-time PCR (qRT-PCR) in paclitaxel-sensitive MDA-MB-468, and paclitaxel-resistant MDA-MB-468R and MDA-MB-231 cells. These results showed that MDA-MB-231 and MDA-MB-468R cells have significantly higher levels of *EDIL3* and *SDC2* than MDA-MB-468 cells. All these data are in concordance with the results obtained by microarray expression, and indicate that *EDIL3* and *SDC2* are overexpressed in paclitaxel-resistant MDA-MB-231 and MDA-MB-468R, as compared with paclitaxel-sensitive MDA-MB-468 cells (Fig. 1d).

### High levels of EDIL3 expression are associated with the mesenchymal phenotype in paclitaxel-resistant cancer cells

**Fig. 2:**
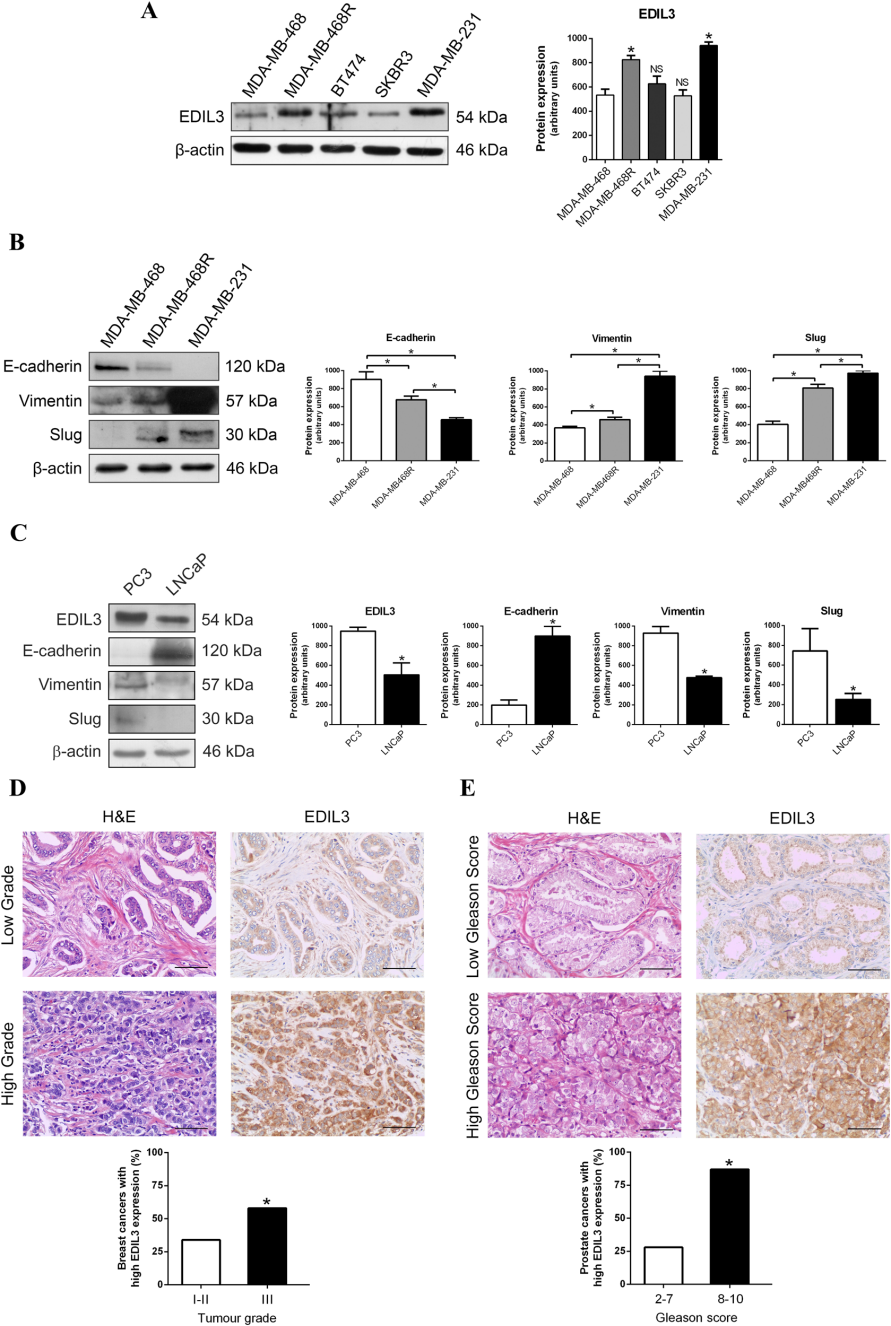
High EDIL3 protein expression is associated with a mesenchymal phenotype in paclitaxel-resistant cancer cells. Western blot analysis of basal expression of EDIL3, and different epithelial and mesenchymal markers are shown. β-actin is shown as a loading control. Histograms show the densitometric analyses of indicated proteins. Data are presented as mean ± SEM (*n* ≥ 3). **a** EDIL3 expression in MDA-MB-468, MDA-MB-468R, BT474, SKBR3, and MDA-MB-231 breast cancer cells. **b** E-cadherin, vimentin, and Slug expression in MDA-MB-468, MDA-MB-468R, and MDA-MB-231 breast cancer cells. **c** EDIL3, E-cadherin, vimentin, and Slug expression in LNCaP and PC3 prostate cancer cells. **d**, **e** Representative micrographs of EDIL3 immunohistochemistry in low- and high-grade breast tumors (**d**), and in low- and high-grade prostate tumors (**e**). Bars, 75 µm. Histograms show percent of breast (**d**) or prostate (**e**) tumors with high EDIL3 expression. Association between EDIL3 and tumor grade was analyzed by Fisher’s exact test. *P < 0.05 from Student’s *t* test; NS not significant.

First, we studied the protein expression levels of EDIL3 in five breast cancer cell lines, which show differences in paclitaxel sensitivity^27^. The highest levels of EDIL3 were observed in MDA-MB-231 cells, followed by MDA-MB-468R, both resistant to paclitaxel; the lowest levels of EDIL3 were observed in SKBR3 cells, followed by MDA-MB-468 and BT474 cells, all of them sensitive to paclitaxel (Fig. 2a). Although different protein expression levels of EDIL3 were observed in these breast cancer cell lines, the statistical analysis showed that when compared with MDA-MB-468 cells, only MDA-MB-468R and MDA-MB-231 cells had a significant increase in EDIL3 protein expression (Fig. 2a).

We chose these three cell lines to examine the protein expression of epithelial marker E-cadherin and mesenchymal markers, vimentin and Slug. MDA-MB-468 cells had the highest levels of E-cadherin, as well as the lowest levels of vimentin and Slug, indicating that this cell line has an epithelial phenotype. Conversely, MDA-MB-231 and MDA-MB-468R cells had the lowest levels of E-cadherin, as well as the highest levels of vimentin and Slug, indicating a mesenchymal phenotype (Fig. 2b).

In summary, paclitaxel-resistant breast cancer cells overexpressed EDIL3 and, surprisingly, these cell lines had a mesenchymal phenotype. To evaluate these results in other types of cancer, we selected paclitaxel-resistant PC3 and paclitaxel-sensitive LNCaP prostate cancer cells. The expression of EDIL3 and mesenchymal markers vimentin and Slug were significantly higher in paclitaxel-resistant PC3 cells than in paclitaxel-sensitive LNCaP cells, while the expression of epithelial marker E-cadherin was higher in LNCaP than in PC3 cells (Fig. 2c). Again, EDIL3 was overexpressed in paclitaxel-resistant cells, which showed a mesenchymal phenotype.

We also performed an immunohistochemical analysis of EDIL3 protein in tumor tissues from 89 breast and 51 prostate cancer patients. Tumor cells showed cytoplasmic immunostaining for this protein. In breast cancer, out of 53 grades I and II breast tumors, 35 (66.0%) expressed low levels of EDIL3, while out of 36 grade III breast tumors, 21 (58.3%) expressed high levels of EDIL3 (Fig. 2d). In prostate cancer, out of 28 low-grade (Gleason ≤ 7) prostate tumors, 20 (71.4%) expressed low levels of EDIL3, while out of 23 high-grade (Gleason 8–10) prostate tumors, 20 (87%) expressed high levels of EDIL3 (Fig. 2e). Statistical analysis of these results showed a significant positive association between tumor grade and EDIL3 expression in breast and prostate cancers (*P* = 0.03 and *P* < 0.0001, respectively).

In addition, we analyzed RNAseq of 843 patients with breast cancer from the TCGA database^28^, and we observe a statistical significance in the group of patients with high levels of mRNA of EDIL3 that had a shorter overall survival at 10 years (Supplementary Fig. S1).

### EDIL3 regulates EMT markers through an autocrine or paracrine mechanism in breast cancer cells

Our results and others point out that EDIL3 acts as an EMT regulator^25^. Since EDIL3 interacts with integrin α_V_β_3_ and other ligands of this integrin are secreted to the ECM and present an autocrine regulation mechanism, we investigated whether EDIL3 was secreted to the medium by breast cancer cells expressing different levels of EDIL3. We also examined the effect of cilengitide, a cyclic pentapeptide whose chemical structure is based on the RGD sequence, which blocks the interaction of EDIL3 with integrin α_V_β_3_ (^29^), on the secretion of EDIL3 and the expression of EMT markers.

**Fig. 3:**
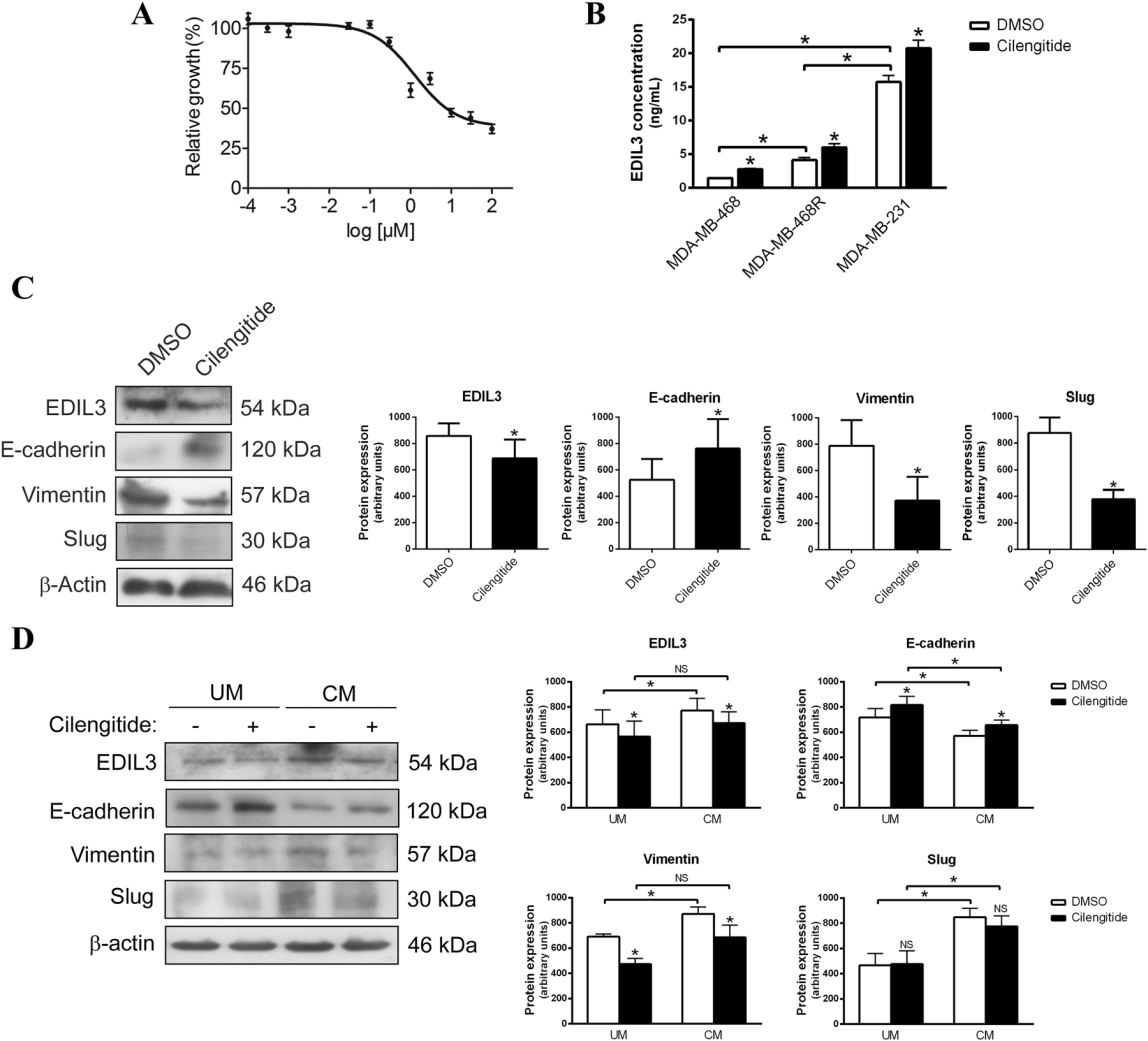
EDIL3 regulates epithelial–mesenchymal transition through an autocrine or paracrine mechanism in breast cancer cells. **a** IC50 curve for cilengitide in MDA-MB-231 breast cancer cell line. Data are presented as mean ± SEM. **b** Histogram shows the concentration of EDIL3 protein in the medium determined by ELISA in MDA-MB-468, MDA-MB-468R, and MDA-MB-231 breast cancer cells treated with DMSO or 1 µM cilengitide during 48 h. **c** Western blot analysis of EDIL3, E-cadherin, vimentin, and Slug in MDA-MB-231 cells treated with DMSO and 1 µM cilengitide during 48 h. β-actin is shown as a loading control. Histograms show the densitometric analyses of indicated proteins. Data are presented as mean ± SEM (*n* ≥ 3). **d** Western blot analysis of EDIL3, E-cadherin, vimentin, and Slug in MDA-MB-468 cells cultured in unconditioned medium (UM) or EDIL3-enriched conditioned medium collected from MDA-MB-231 cells (CM), and treated with DMSO and 1 µM cilengitide during 48 h. β-actin is shown as a loading control. Histograms show the densitometric analyses of indicated proteins. Data are presented as mean ± SEM (*n* ≥ 3). *P < 0.05 from Student’s *t* test; NS not significant.

To choose the optimal concentration of cilengitide, we determined the half inhibitory concentration (IC50) for this agent in MDA-MB-231 cells because this cell line had the highest levels of EDIL3. MDA-MB-231 cells were treated with different concentrations of cilengitide from 10^−4^ to 10^2^ µM during 72 h, and cell viability was evaluated. The IC50 of cilengitide was calculated as 1.25 ± 0.04 µM in this breast cancer cell line (Fig. 3a), so subsequent experiments were carried out with 1 µM cilengitide.

MDA-MB-468, MDA-MB-468R, and MDA-MB-231 cells were treated with dimethyl sulfoxide (DMSO) or 1 µM cilengitide during 48 h, and the concentration of secreted EDIL3 was measured by enzyme-linked immunosorbent assay (ELISA). Our results demonstrated that the three cell lines secreted EDIL3, with the lowest concentration of secreted EDIL3 observed in paclitaxel-sensitive MDA-MB-468 cells, and the highest in paclitaxel-resistant MDA-MB-468R and MDA-MB-231 cells, similar to the intracellular EDIL3 expression levels reported in these cells. Interestingly, after cilengitide treatment, the concentration of secreted EDIL3 increased from 1.46 ± 0.12 to 2.76 ± 0.22 ng/ml in MDA-MB-468 cells, from 4.13 ± 0.63 to 6.03 ± 0.93 ng/ml in MDA-MB-468R cells, and from 15.73 ± 1.67 to 20.73 ± 2.12 ng/ml in MDA-MB-231 cells (Fig. 3b). We also observed that increased levels of secreted EDIL3 after cilengitide treatment were accompanied by the upregulation of E-cadherin, and the downregulation of intracellular EDIL3 and vimentin in MDA-MB-231 and MDA-MB-468 cells, and Slug in MDA-MB-231 cells (Fig. 3c, d). These results suggest that extracellular levels of EDIL3 and its involvement in EMT regulation could be mediated by an autocrine or paracrine mechanism.

To further investigate this hypothesis, we examined the effect of extracellular EDIL3 in MDA-MB-468 cells using EDIL3-enriched conditioned medium (CM) collected from MDA-MB-231 cells. MDA-MB-468 cells growing in CM showed higher expression of EDIL3, vimentin, and Slug, and lower expression of E-cadherin in comparison with MDA-MB-468 growing in unconditioned medium (UM), and this trend reverted when MDA-MB-468 growing in CM were treated with cilengitide (Fig. 3d). Furthermore, an immunohistochemical analysis of E-cadherin was performed to confirm this hypothesis (Supplementary Fig. S2). Together these results confirm that extracellular EDIL3 regulates EMT through an autocrine or paracrine mechanism, involving its interaction with integrin α_V_β_3_.

### EDIL3 gene silencing increases paclitaxel-induced apoptosis and reverts EMT in paclitaxel-resistant cancer cells

**Fig. 4:**
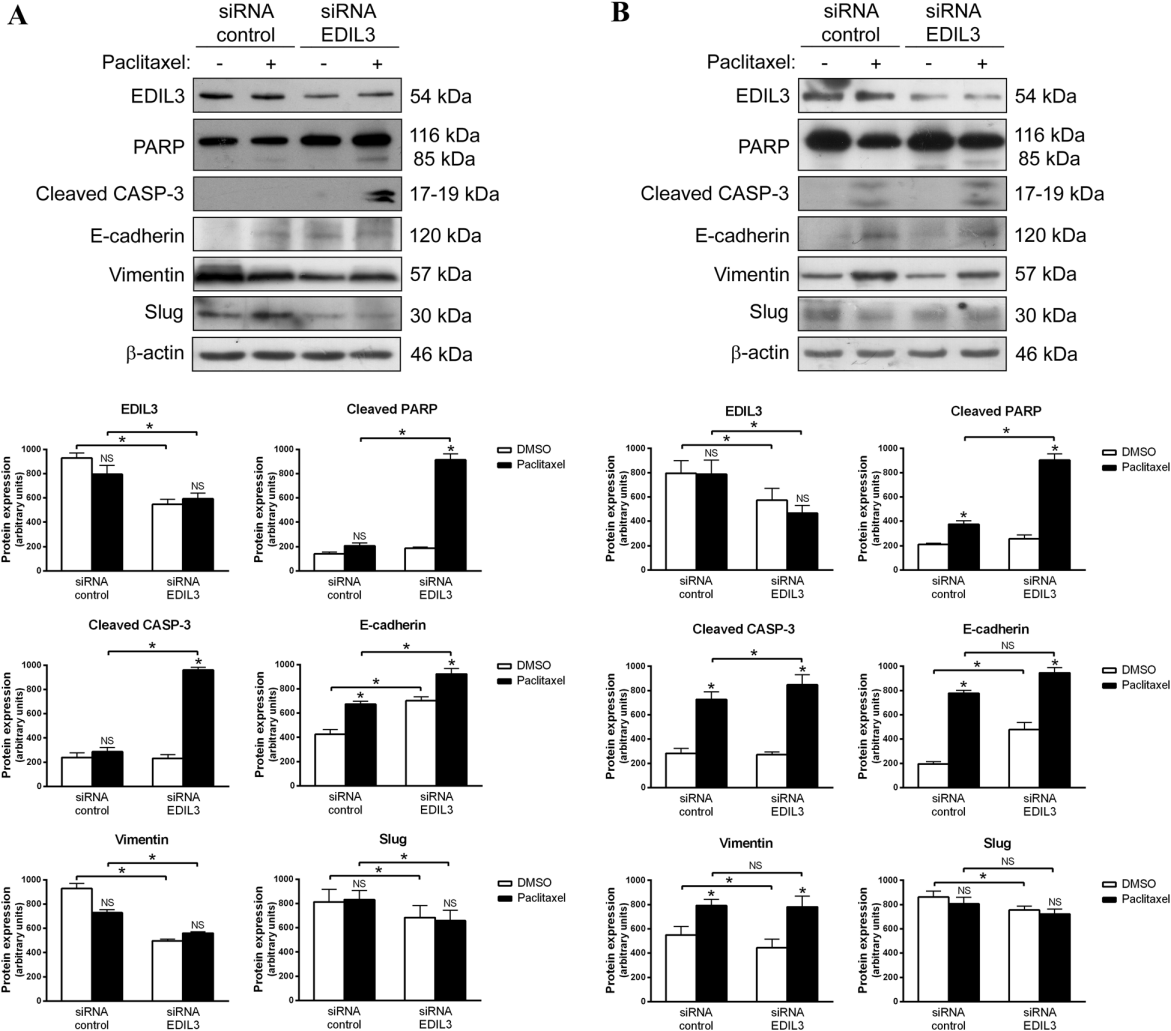
EDIL3 gene silencing increases paclitaxel-induced apoptosis and reverts epithelial–mesenchymal transition in paclitaxel-resistant cancer cells. **a** MDA-MB-231 and **b** PC3 cells were silenced for EDIL3, and treated with DMSO and 1 or 2.5 µM paclitaxel, respectively. Paclitaxel-induced apoptosis was assessed by western blot analysis of cleaved PARP and active caspase-3. Modulation of EDIL3, E-cadherin, vimentin, and Slug was studied by western blot analysis, using β-actin as a loading control. Histograms show the densitometric analyses of indicated proteins. Data are presented as mean ± SEM (*n* ≥ 3). *P < 0.05 from Student’s *t* test; NS not significant.

Because the paclitaxel-resistant MDA-MB-231 and PC3 cell lines presented the highest levels of EDIL3 and a mesenchymal phenotype, they were selected to gain insight into the involvement of EDIL3 in paclitaxel resistance and EMT. For that, EDIL3 expression was silenced using a specific pool of small interfering RNA (siRNA), and breast and prostate cancer cells were subsequently treated with 1 or 2.5 µM paclitaxel during 48 h, respectively. EDIL3-silenced MDA-MB-231 cells showed increased PARP cleavage and caspase-3 activation after paclitaxel treatment, as compared with siRNA control cells (Fig. 4a). We also confirm the role of EDIL3 in the paclitaxel resistance with a long-term viability assay in this cell line (Supplementary Fig. S3a). With respect to EMT markers, EDIL3-silenced MDA-MB-231 cells showed increased expression of E-cadherin, while the expression of vimentin and Slug were decreased as compared with siRNA control cells (Fig. 4a). Although paclitaxel treatment induced the upregulation of E-cadherin levels, especially in EDIL3-silenced MDA-MB-231 cells, the levels of vimentin or Slug were not significantly affected by paclitaxel treatment (Fig. 4a).

In the case of PC3 cells, the induction of apoptosis was more potent in EDIL3-silenced PC3 cells as evidenced by the increase in cleaved PARP and active caspase-3 after paclitaxel treatment, as compared with siRNA control cells (Fig. 4b). Regarding the EMT markers, EDIL3 gene silencing induced the downregulation of vimentin and Slug, as well as the upregulation of E-cadherin. Although an increase in E-cadherin and vimentin was observed after paclitaxel treatment, statistically significant differences were not observed between paclitaxel-treated siRNA control PC3 cells and paclitaxel-treated EDIL3-silenced PC3 cells (Fig. 4b). The invasion ability of PC3 cells was tested in a Matrigel assay, in which was confirmed that the downregulation of endogenous EDIL3 expression decreases the invasive potential of PC3 cells (Supplementary Fig. S3b).

### Extracellular EDIL3 or EDIL3 overexpression favors EMT and reduces paclitaxel sensitivity in cancer cells

**Fig. 5:**
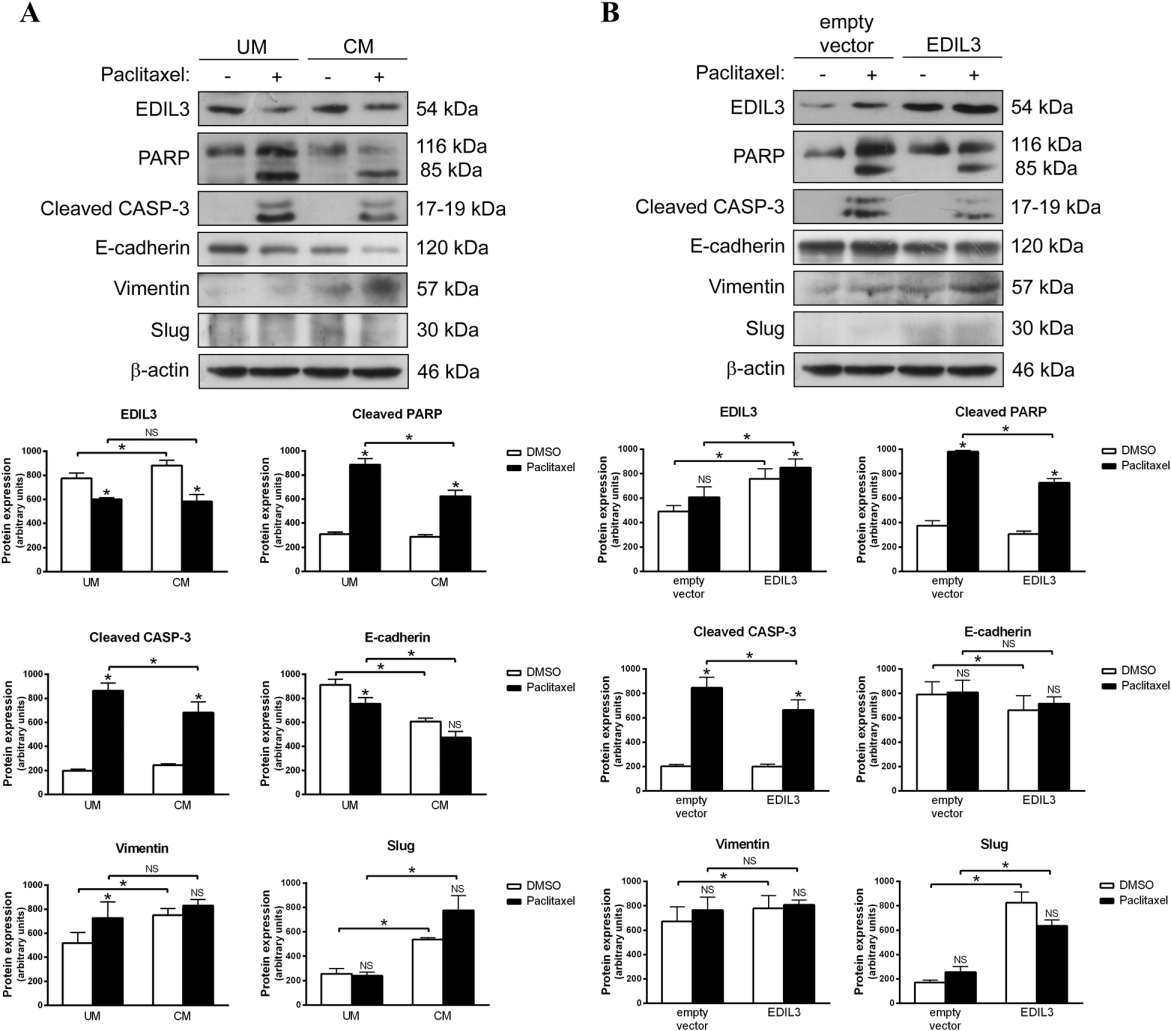
Extracellular EDIL3 and EDIL3 overexpression promotes epithelial–mesenchymal transition and paclitaxel resistance in cancer cells. **a** MDA-MB-468 breast cancer cells were cultured in unconditioned medium (UM) or EDIL3-enriched conditioned medium collected from MDA-MB-231 cells (CM), and treated with DMSO and 1 µM paclitaxel during 48 h. **b** LNCaP prostate cancer cells were transiently transfected with pCMV6-XL4-EDIL3 or with empty vector, and treated with DMSO or 2.5 µM paclitaxel during 48 h. Paclitaxel-induced cleavage of PARP and caspase-3, as well as paclitaxel-induced modulation of EDIL3, E-cadherin, vimentin, and Slug were analyzed by western blot using β-actin as a loading control. Histograms represent the densitometric analyses of indicated proteins. Data are presented as mean ± SEM (*n* ≥ 3). *P < 0.05 from Student’s *t* test; NS, not significant.

As paclitaxel-sensitive MDA-MB-468 and LNCaP cells presented the lowest levels of EDIL3 and an epithelial phenotype, they were selected to study the effect of extracellular EDIL3 or EDIL3 overexpression on the response to paclitaxel and in the regulation of EMT. The effect of extracellular EDIL3 was examined using CM collected from MDA-MB-231 cells and used to culture MDA-MB-468 cells, subsequently treated with DMSO or 1 µM paclitaxel during 48 h. MDA-MB-468 cells growing in CM showed more resistance to paclitaxel-induced apoptosis than MDA-MB-468 cells cultured in UM, as indicated by the lower levels of cleaved PARP and caspase-3 (Fig. 5a). This was also verified by annexin V binding assays (Supplementary Fig. S4). Furthermore, additional experiments using CM from EDIL3 and control siRNA-treated MD-MB-231 cells showed that paclitaxel sensitivity was restored in MDA-MB-468 cells grown in siRNA EDIL3 CM, both by western blot and annexin V binding assays (Supplementary Figs. S5a, b and S6). Together these results further confirm that extracellular EDIL3 regulates paclitaxel response in breast cancer cells.

Also, cells cultured in CM showed a more mesenchymal phenotype than cells cultured in UM. Although no effect on Slug expression was observed after paclitaxel treatment, the downregulation of EDIL3 and E-cadherin, as well as the upregulation of vimentin were observed after paclitaxel treatment in MDA-MB-468 cells growing in UM vs. CM (Fig. 5a).

To further investigate the role of EDIL3 in paclitaxel response and EMT regulation, paclitaxel-sensitive LNCaP cells were transiently transfected with pCMV6-XL4-EDIL3, and treated with DMSO or 2.5 µM paclitaxel during 48 h. The cleavage of PARP and the activation of caspase-3 were lower in paclitaxel-treated EDIL3-transfected cells than in paclitaxel-treated empty vector-transfected cells (Fig. 5b). Contrary to the EDIL3 gene silencing results, EDIL3 overexpression resulted in decreased E-cadherin levels, and increased vimentin and Slug levels, with no changes observed in the expression of these proteins after paclitaxel treatment (Fig. 5b). Overall, these results suggest that EDIL3 regulates the paclitaxel response and EMT in both breast and prostate cancer cells via an autocrine or paracrine mechanism.

### Cilengitide sensitizes paclitaxel-resistant cancer cells to paclitaxel-induced apoptosis and reverts the expression of EMT markers

Cilengitide has shown a modest improvement in overall survival and progression-free survival in a phase II clinical trial, and a trend of benefit in overall survival in a phase III clinical trial enrolling glioblastoma patients^30^. Moreover, a phase I clinical trial is currently testing the efficacy of cilengitide in combination with paclitaxel in patients with solid tumors^31^. So, we decided to investigate whether the blockade of the interaction between EDIL3 and integrin α_V_β_3_ with cilengitide had an effect on the paclitaxel response of cancer cells. To perform these experiments, paclitaxel-resistant MDA-MB-231 and PC3 cells were treated with DMSO, 1 µM cilengitide, 1 or 2.5 µM paclitaxel, and the combination of 1 µM cilengitide and 1 or 2.5 µM paclitaxel, respectively, during 48 h.

**Fig. 6:**
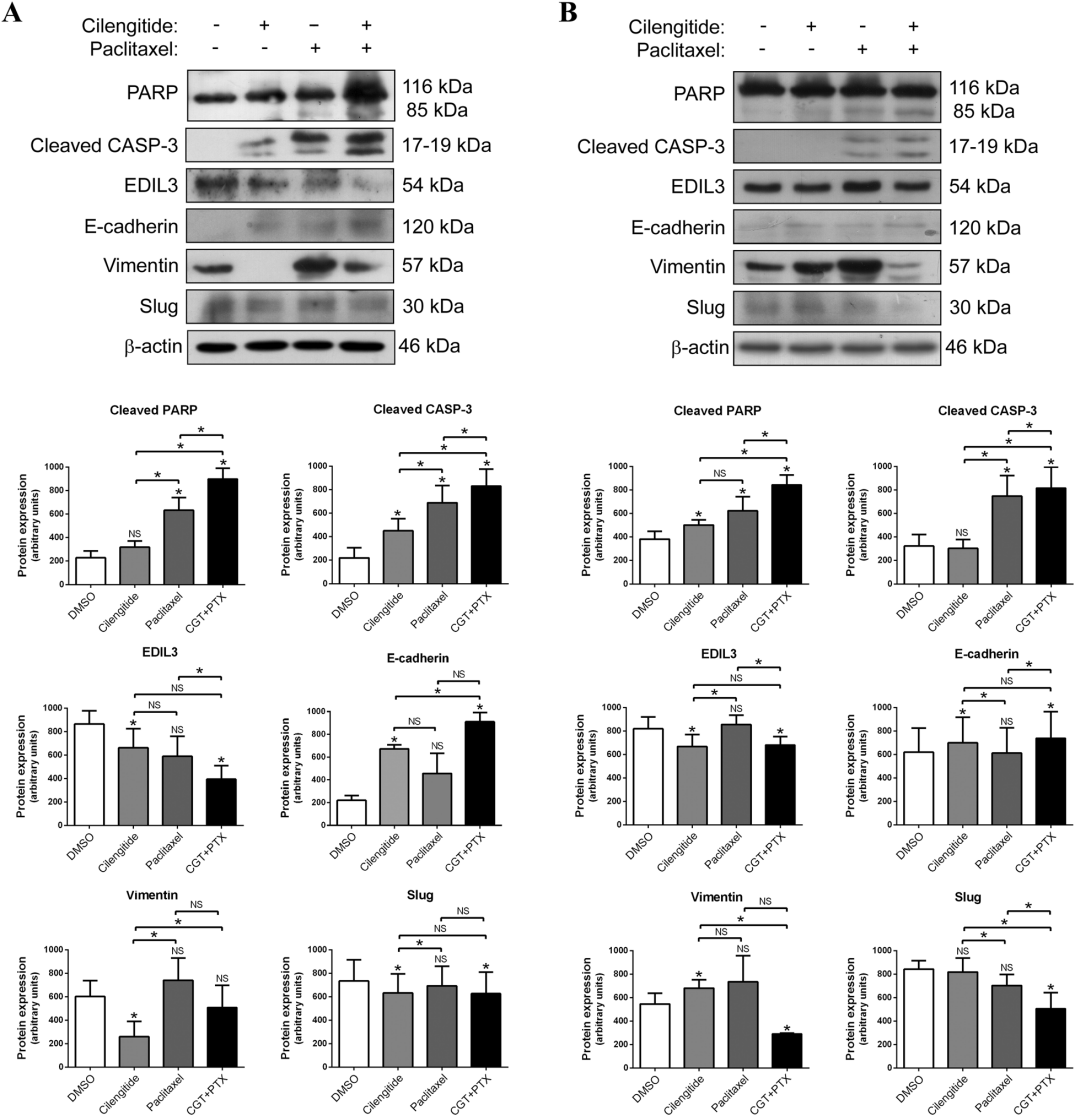
Cilengitide sensitizes paclitaxel-resistant cancer cells to paclitaxel-induced apoptosis and reverts mesenchymal transition. **a** MDA-MB-231 and **b** PC3 cells were treated with DMSO, 1 µM cilengitide, 1 or 2.5 µM paclitaxel, and 1 µM cilengitide plus 1 or 2.5 µM paclitaxel, respectively. The induction of apoptosis was studied through the cleavage of PARP and caspase-3 by western blot, as well as the expression of indicated epithelial–mesenchymal transition markers. β-actin was used as a loading control. Histograms show the densitometric analyses of indicated proteins. Data are presented as mean ± SEM (*n* ≥ 3). *P < 0.05 from Student’s *t* test; NS not significant.

As observed by PARP cleavage and caspase-3 activation, the combined treatment of MDA-MB-231 or PC3 cells with cilengitide and paclitaxel resulted in a greater induction of apoptosis than with either agent alone (Fig. 6a, b). This was also verified by annexin V binding assays (Supplementary Fig. S7). Combined treatment also induced the upregulation of E-cadherin and the downregulation of EDIL3, vimentin, and Slug in comparison with DMSO-treated MDA-MB-231 and PC3 cells (Fig. 6a, b). In the case of MDA-MB-231 cells, cilengitide treatment upregulated E-cadherin and downregulated the mesenchymal markers EDIL3, vimentin, and Slug (Fig. 6a). Similarly, cilengitide induced the downregulation of EDIL3 and the upregulation of E-cadherin in PC3 cells; whereas vimentin expression was increased and no change was observed for Slug expression after cilengitide treatment (Fig. 6b). In summary, these results demonstrate that cilengitide sensitizes paclitaxel-resistant cancer cells to paclitaxel-induced apoptosis, and that this sensitization is associated with modulation of the EMT process.

## Discussion

Breast and prostate cancers are the most frequent cancers among women and men, respectively^12,21,25^. Taxanes are incorporated into several adjuvant chemotherapy regimens and have demonstrated efficacy in the treatment of cancer. However, many patients develop drug resistance, resulting in cancer recurrence and metastasis. Thus, it is important to analyze the molecular mechanisms of paclitaxel resistance and search for new predictive molecular markers, as a strategy for the establishment of safer and more effective therapeutic regimens^26^. We observed that several EMT-related genes were differentially expressed in paclitaxel-resistant in comparison with paclitaxel-sensitive breast cancer cells. Among others, we observed the upregulation of *EDIL3*, *SPOCK1*, and *SDC2*, and the downregulation of *TET2* in paclitaxel-resistant cells. EDIL3 is essential in angiogenesis and vascular development^13,14,16,32^, but it is also involved in the promotion of migration, invasion, and EMT^20,25,33^. Recently, EDIL3 expression has been observed in several tumor types, including breast cancer, in which this protein has been identified as a biomarker for early disease detection^21,25^. In this study, we demonstrated a significant association between EDIL3 protein expression and Gleason score in prostate cancer. We also found an association between EDIL3 protein expression and tumor grade in breast cancer, as previously reported^23^, and to explore the correlation between EDIL3 expression and overall survival of the patients, we studied the TCGA database where we analyzed the RNAseq in breast cancer patients^28^. We observed a statistical significance in the group of patients with high levels of mRNA of EDIL3 that had a shorter overall survival at 10 years, in a Kaplan–Meier’s survival curve. SDC2 is involved in the tissue development and homeostasis, but its overexpression has also been described in various types of cancers, including breast cancer, favoring a more aggressive phenotype by the inhibition of apoptosis and promotion of cell migration, invasion, and metastasis^34–37^.

These observations were also validated in MDA-MB-231 paclitaxel-resistant breast cancer cells, where higher levels of *EDIL3* and *SDC2* indicated the involvement of the EMT process in paclitaxel resistance, as reported in ovarian cancer^38,39^. Similarly, Zhang et al. described the acquisition of mesenchymal properties in breast cancer cells after docetaxel treatment, and showed that this conferred greater tumorigenic and metastatic potential in mice^40^. Other studies support the premise that EMT allows epithelial breast cancer cells to metastasize to other organs, suggesting that EMT-related genes play an important role in the progression, metastasis, and recurrence of breast cancer^41^. In relation to prostate cancer, androgen deprivation therapy is temporarily effective, but it can simultaneously induce EMT, and this may lead to castration-resistant progression. Moreover, EMT induction correlates with high Gleason grade, poor clinical behavior of the disease, and with the docetaxel resistance of prostate cancer cells in vitro^11,12,42^.

We observed high levels of EDIL3 protein in paclitaxel-resistant breast and prostate cancer cells that also showed a mesenchymal phenotype. When the interaction of EDIL3 with integrin α_V_β_3_ was blocked using cilengitide, this mesenchymal phenotype was reverted. Furthermore, the recovery of the EMT in low EDIL3-secreting cells grown in the CM of high EDIL3-secreting cells, and the loss of this process with subsequent cilengitide treatment added weight to the hypothesis that both extracellular EDIL3 levels and the regulation of EMT by EDIL3 are mediated through an autocrine or paracrine mechanism in cancer cells. Concordantly, Feng et al. described an autocrine regulation of EDIL3 and its contribution to a receptive microenvironment for the detachment of hepatocellular carcinoma cells from the primary lesion and the promotion of resistance to anoikis^43^. Our experiments also associated EDIL3 and EMT with paclitaxel resistance, since EDIL3 gene silencing sensitized cells to paclitaxel-induced apoptosis and reversed the EMT process. In addition, paclitaxel-sensitive cancer cells overexpressing-EDIL3 or cultured in CM were more resistant to this drug, and showed the induction of EMT. Therefore, our data suggest that EDIL3 is a key regulator of EMT, and determines sensitivity to paclitaxel in breast and prostate cancer cells.

Although there is controversy with respect to the apoptotic effect of cilengitide, this drug inhibits cell growth in vitro and tumor growth, angiogenesis and bone metastases in vivo^43–46^. Moreover, its combination with radiotherapy or chemotherapy increases the therapeutic efficiency^47–49^, and a current phase I clinical trial is testing the combination of cilengitide and paclitaxel in patients with solid tumors^31^. We show that combined treatment caused a greater apoptosis induction than either drug alone, and this was associated with the reversion of the mesenchymal phenotype in paclitaxel-resistant cancer cell lines. These results support the design of therapeutic strategies based on the combination of cilengitide and paclitaxel for the treatment of patients, with advanced breast or prostate cancer.

In conclusion, we show that high levels of EDIL3 play a prominent role in EMT, as well as in paclitaxel resistance in both breast and prostate cancer cells. The blockade of the interaction of secreted EDIL3 with the integrin α_V_β_3_ by cilengitide restored sensitivity to paclitaxel, and reverted the mesenchymal phenotype in paclitaxel-resistant cancer cells. These findings provide the basis for future studies to determine the diagnostic value of EDIL3 and the efficacy of combined treatment with cilengitide and paclitaxel, as a new therapeutic strategy in patients with breast and prostate cancer.

## Materials and methods

### Cell culture

MDA-MB-231, MDA-MB-468, SKBR3, and BT474 breast cancer cell lines, and PC3 and LNCaP prostate cancer cell lines from the Interlab Cell Line Collection (Genoa, Italy) were routinely grown in RPMI 1640 (Lonza, Basel, Switzerland) supplemented with 10% fetal bovine serum (FBS; Sigma, St Louis, MO, USA), 50 U/ml penicillin, 50 µg/ml streptomycin (Sigma), 10 mM HEPES buffer (Lonza), and 1 mM glutamine (Lonza) in a 37 °C humidified incubator under 5% CO_2_. Subconfluent cell cultures were harvested by trypsinization. All the experiments were performed using cells that had not exceeded the first ten passages after receipt of the initial vial, and were routinely tested for *Mycoplasma* contamination. The paclitaxel-resistant MDA-MB-468R cell line was generated in our laboratory as previously described^27^. All cell lines were authenticated by SRT profiling (qGenomics, August 29^th^ 2019).

### Apoptosis induction assays

Stock solutions of paclitaxel (Calbiochem, San Diego, CA, USA) and cilengitide (Selleck Chemicals, Houston, TX, USA) were prepared at 10 mM in DMSO (Sigma) and stored at −20 °C. DMSO was added to untreated cells. For combined treatments, both drugs were added simultaneously during 48 h. Caspase activation and cleavage of PARP were assessed by western blotting to determine the induction of apoptosis.

### Annexin V binding assays

Subconfluent monolayers of cells were treated with their corresponding drug for 48 h. One million cells were washed in cold phosphate-buffered saline (PBS), and suspended in 100 µl annexin V binding buffer containing 5 µl propidium iodide and 5 µl annexin V-FITC (Annexin V-FITC Apoptosis Detection Kit I, BD Biosciences, San Jose, CA, USA), incubated for 15 min at room temperature in the dark, and diluted in 400 µl annexin V binding buffer. Fluorescence was measured on a FACSCanto II cytometer (BD Biosciences) within 1 h. Cell populations (viable, early apoptotic, and late apoptotic) were identified measuring fluorescence on FITC-A and PerCP-Cy5-5-A channels. Annexin V binding assays were repeated three times in independent experiments.

### Differential expression analysis

Total RNA from MDA-MB-468 and MDA-MB-468R cells was isolated using RNeasy Micro Kit (Qiagen, Hilden, Germany) according to the manufacturer’s instructions. The genome expression profile was analyzed using Affymetrix Human Gene 1.0 ST Array (Affymetrix Thermo Fisher Scientific, Santa Clara, CA, USA), which includes 32,321 probe sets. Differential expression between MDA-MB-468 and MDA-MB-468R cells was assessed using Student’s *t* test and the Benjamini–Hochberg false discovery rate correction for multiple testing^50^. Results were considered significant when adjusted *P* values were <0.05, and only those showing a fold change ≥ 2 between MDA-MB-468 and MDA-MB-468R cells were considered for further meta-analysis. Raw intensity data values from parental MDA-MB-231 and paclitaxel-resistant RMDA-MB-231 cells using the Affymetrix Human Genome U133A Array were retrieved from the GEO repository (E-GEOD-12791) and analyzed as described for MDA-MB-468 cells. MetaDE R package was used to perform a meta-analysis between both studies using a fixed effect model.

### Reverse transcription and quantitative real-time PCR

Total RNA was extracted from cells using the AllPrep DNA/RNA/miRNA Universal kit (Qiagen). Using SuperScript III and oligo (dT) primer (Invitrogen, Thermo Fisher Scientific, Carlsbad, CA, USA), according to the manufacturer’s instructions, 1 μg RNA was reverse-transcribed to prepare the cDNA samples for qRT-PCR. Taqman Gene Expression Master Mix and Assays Probes (Applied Biosystems, Thermo Fisher Scientific, Foster City, CA, USA) were used for qRT-PCR analyses of *EDIL3*, *SDC2*, *TBP*, and *EIF2B2* genes. StepOne Real-Time PCR System (Applied Biosystems, Thermo Fisher Scientific) was used and reactions were run in triplicates. The expression was calculated with the relative quantification method 2^−ΔCT^, in which expression of *EDIL3* and *SDC2* genes was assessed relative to the expression of the house-keeping genes *TBP* and *EIF2B2*.

### Small interfering RNA

RNA interference was carried out using DharmaFECT 2 reagent (Dharmacon, Lafayette, CO, USA) according to manufacturer’s instructions. Validated pools of EDIL3-siRNA and non-targeting siRNA as negative control were ordered from Dharmacon (ON-TARGETplus SMART pools L-017593 and D-001810) and used at 50 nM. Cells were subjected to drug treatments 24 h after RNA interference.

### Plasmid transfections

Transient transfections were carried out using the FuGENE reagent (Promega, Madison, WI, USA) according to the manufacturer’s instructions. pCMV6-XL4-EDIL3 and pCMV6-XL4 plasmids were obtained from OriGene (Rockville, MD, USA). Cells were subjected to drug treatments 24 h after transfection.

### Antibodies

The antibodies and dilutions used for western blots were as follows: rabbit polyclonal anti-EDIL3 (1:1000, cat #PA5-27994, Thermo Fisher Scientific, Waltham, MA, USA), mouse monoclonal anti-β-actin (1:20000, cat #A5441, Sigma), rabbit polyclonal anti-cleaved caspase-3 (1:500, cat #9664, Cell Signaling Technology, Danvers, MA, USA), mouse monoclonal anti-Slug (1:1000, cat #166476, Santa Cruz Biotechnology, Santa Cruz, CA, USA), mouse monoclonal anti-PARP (1:500, cat #551024, BD Biosciences), mouse monoclonal anti-E-cadherin (1:5000; cat #610181, BD Biosciences), and rabbit monoclonal anti-vimentin (1:1000, cat #92547, Abcam, Cambridge, UK).

### Western blotting

Cells were lysed in Nonidet P-40 (NP40) lysis buffer (10 mM Tris-HCl pH 7.5, 150 mM NaCl, 10% glycerol, and 1% NP40). Equal amounts of total protein, as determined by the BCA Protein Assay Kit (Thermo Scientific, Pierce, Rockford, IL, USA), were separated by SDS–PAGE on 8% polyacrylamide gels and electroblotted onto nitrocellulose membranes (GE Healthcare, Little Chalfont, UK). Membranes were stained with Ponceau S to ensure that protein amounts were comparable. For immunodetection, blots were blocked in 1% blocking reagent (Roche, Mannheim, Germany) in 0.05% Tween 20-PBS for 1 h and incubated with primary antibody diluted in blocking buffer overnight at 4 °C. Blots were then washed in 0.05% Tween 20-PBS and incubated with either goat anti-mouse (1:20000; GE Healthcare) or goat anti-rabbit (1:20000; GE Healthcare) peroxidase-labeled antibodies in blocking buffer for 1 h. The enhanced chemiluminescent system was applied according to the manufacturer’s protocol (GE Healthcare). Chemiluminescent signal was detected on Amersham Hyperfilm^TM^-ECL (GE Healthcare) at different exposure times. The experiments were performed at least three times. Densitometric analysis was performed using QuantiScan software (Biosoft, Cambridge, UK). Arbitrary densitometric units of the proteins of interest were corrected against β-actin. Data comparing differences between two conditions were statistically analyzed using Student’s *t* test. Statistical analyses were performed with Prism 6.0 software (GraphPad, San Diego, CA, USA). Differences were considered as significant when *P* < 0.05.

### Cell viability assay

AlamarBlue Cell Viability Assay Reagent (Thermo Fisher Scientific) was used according to manufacturer’s instructions to determine the IC50 for cilengitide in MDA-MB-231 cells. Briefly, 10000 cells in exponential cell growth were plated in 96-well plates (Nunc, Thermo Fisher Scientific, Roskilde, Denmark). After cell adherence, cells were treated with serial dilutions of cilengitide to span the dose range suitable from 0.0001 to 100 µM during 72 h in triplicate. Fluorescence was measured with FLx800 Microplate Fluorescence Reader (Bio-Tek, Winooski, VT, USA). Data were normalized to the vehicle treatment and IC50 was calculated using Prism 6.0 software.

### Enzyme-linked immunosorbent assay

MDA-MB-468, MDA-MB-468R, and MDA-MB-231 cells were grown in six-well plates until 90% sub-confluence in FBS-free RPMI 1640 medium, and treated with DMSO or cilengitide during 72 h. Media from each condition were taken to measure secreted EDIL3. ELISA kit for EDIL3 (Cloud Clone, Katy, TX, USA) was used according to the manufacturer’s protocol. For each ELISA assay, analyses were performed in triplicate and the experiments were repeated at least three times. Absorbance was measured at 450 nm and values were analyzed by Prism 6.0 software.

### Conditioned media

MDA-MB-231 cells were cultured in supplemented RPMI 1640 medium. When cultures were ~70% confluent, CM was collected, centrifuged at 3000 r.p.m. for 10 min, and stored at 4 °C during at least 72 h before use. To assay the effects of CM, MDA-MB-468 cells were cultured in supplemented RPMI 1640 medium or in CM collected from MDA-MB-231, and subjected to different treatments after 48 h. In addition, conditioned media were collected from EDIL3 and control siRNA-treated MDA-MB-231 cells after 48 h (siRNA EDIL3 CM and siRNA control CM, respectively).

### Long-term viability assay

MDA-MB-231 cells were seeded in 12-well plate and transfected with siRNA control or siRNA EDIL3. After 24 h, cells were treated with DMSO or paclitaxel 1 µM for 48 h and in that moment, the medium with drugs was removed and replaced with fresh medium. Upon 35 days, cells were fixed and stained with Quick Panoptic (QCA, Tarragona, Spain). The presence of healthy nuclei was evaluated, and statistical analysis was performed with the percentage of healthy nuclei respect to the total nuclei.

### Tumor cell invasion assay

The invasion chamber consists of a 24-well plate with Matrigel inserts (BD Biosciences) containing an 8-lm pore-size PET membrane coated with a thin layer of ECM. PC3 cells silenced during 24 h were trypsinized and suspended in serum-free RPMI 1640, and added to the upper chamber at 2 × 10^4^ cells/insert. The lower chamber was filled with medium containing 5% FBS as chemoattractant. After 48 h of culture, the upper surface of the inserts was wiped with cotton swabs, and the inserts were stained with Quick Panoptic (QCA) and evaluated under light microscopy.

### Patients and tumor immunohistochemistry

Formalin-fixed paraffin-embedded tissues from 89 breast cancer patients and 51 prostate cancer patients were selected for the assembly of tissue microarrays, with the approval of the institution’s ethical board (CEI Hospital Universitario Virgen del Rocío 0096-N-18-33180005). Tissue sections of 5 µm were dewaxed, rehydrated, and immersed in 3% H_2_O_2_ aqueous solution for 30 min to exhaust endogenous peroxidase. Antigen retrieval was performed with 1 mM EDTA (pH 9.0) and sections were incubated overnight at 4 °C with anti-EDIL3 antibody (1:75). Peroxidase-labeled secondary antibodies and 3,3′ diaminobenzidine were applied according to the manufacturer’s protocol (EnVision, Dako, Agilent Technology, Glostrup, Denmark). Slides were counterstained with hematoxylin and mounted. Immunostains were scored as low (<10%) or high expression (≥10%) according to the extent of positive cells. Correlation between EDIL3 expression and tumor grade were analyzed by Fisher’s exact test using SPSS software (IBM, Armonk, NY, USA). Differences were considered as significant when *P* < 0.05.

## Supplementary information

Supplementary Figure S1-20-0879R

Supplementary Figure S2-20-0879R

Supplementary Figure S3-20-0879R

Supplementary Figure S4-20-0879R

Supplementary Figure S5-20-0879R

Supplementary Figure S6-20-0879R

Supplementary Figure S7-20-0879R

Supplementary Table S1-20-0879R

Supplementary Table S2-20-0879R

Supplementary Table S3-20-0879R
