# Supplementary figures and images for: EDIL3 promotes epithelial–mesenchymal transition and paclitaxel resistance through its interaction with integrin αVβ3 in cancer cells
Source: Cell Death Discov. 2020 Sep 16;6:86. doi: 10.1038/s41420-020-00322-x (PMC7494865; doi:10.1038/s41420-020-00322-x)

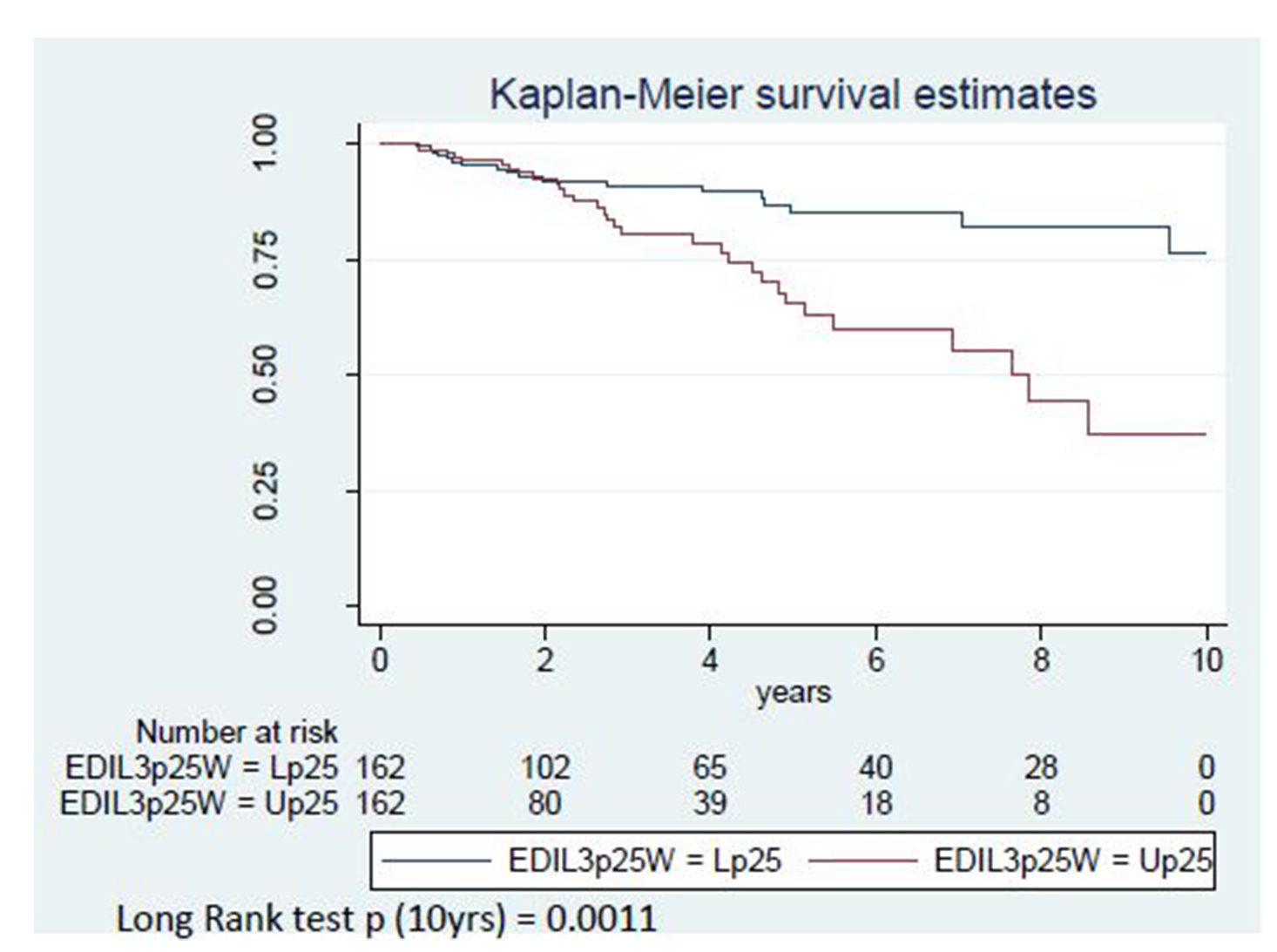

Supplement: Supplementary file 1 — Supplementary Figure S1-20-0879R [file 41420_2020_322_MOESM1_ESM.tif]

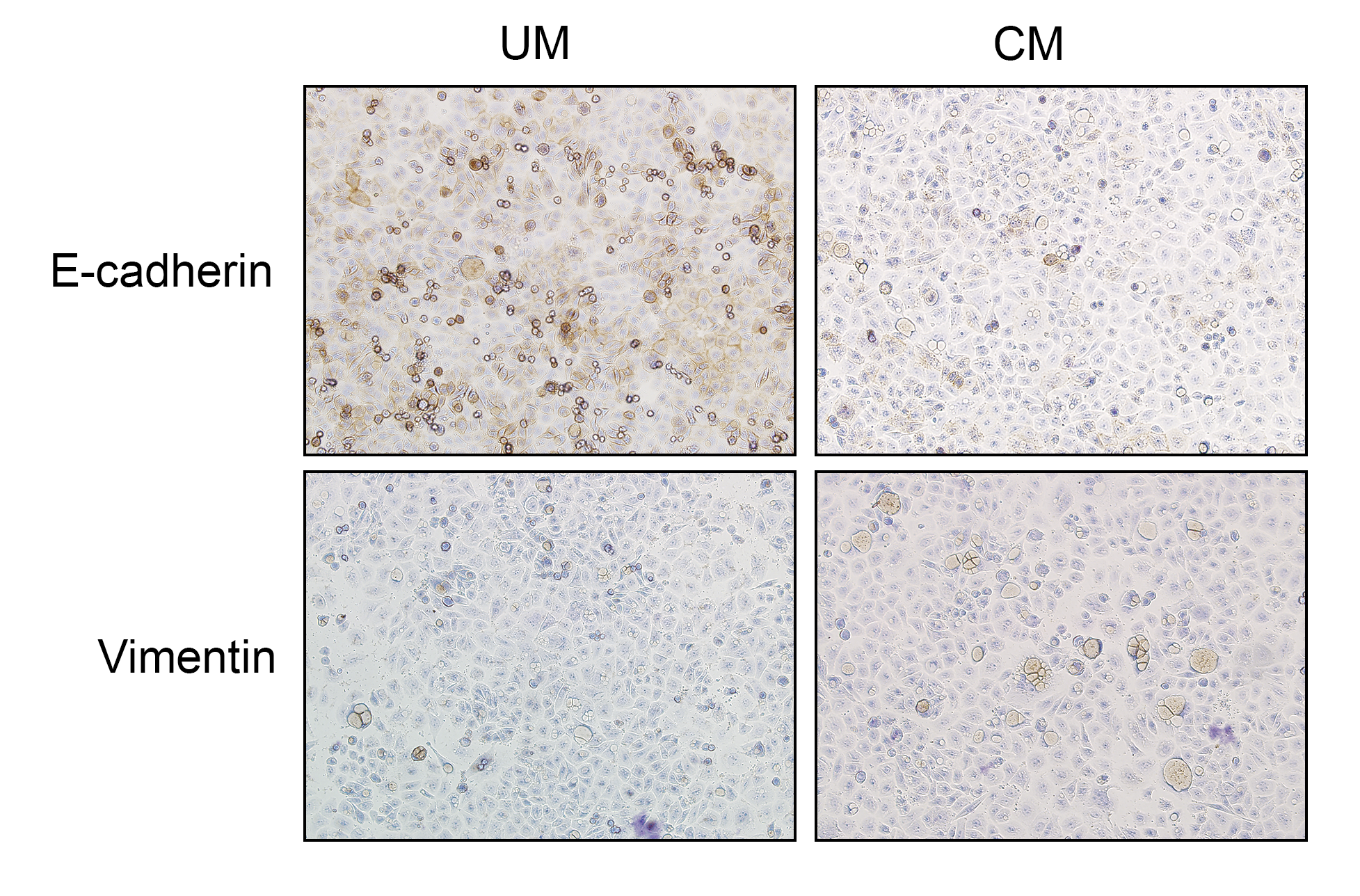

Supplement: Supplementary file 2 — Supplementary Figure S2-20-0879R [file 41420_2020_322_MOESM2_ESM.tif]

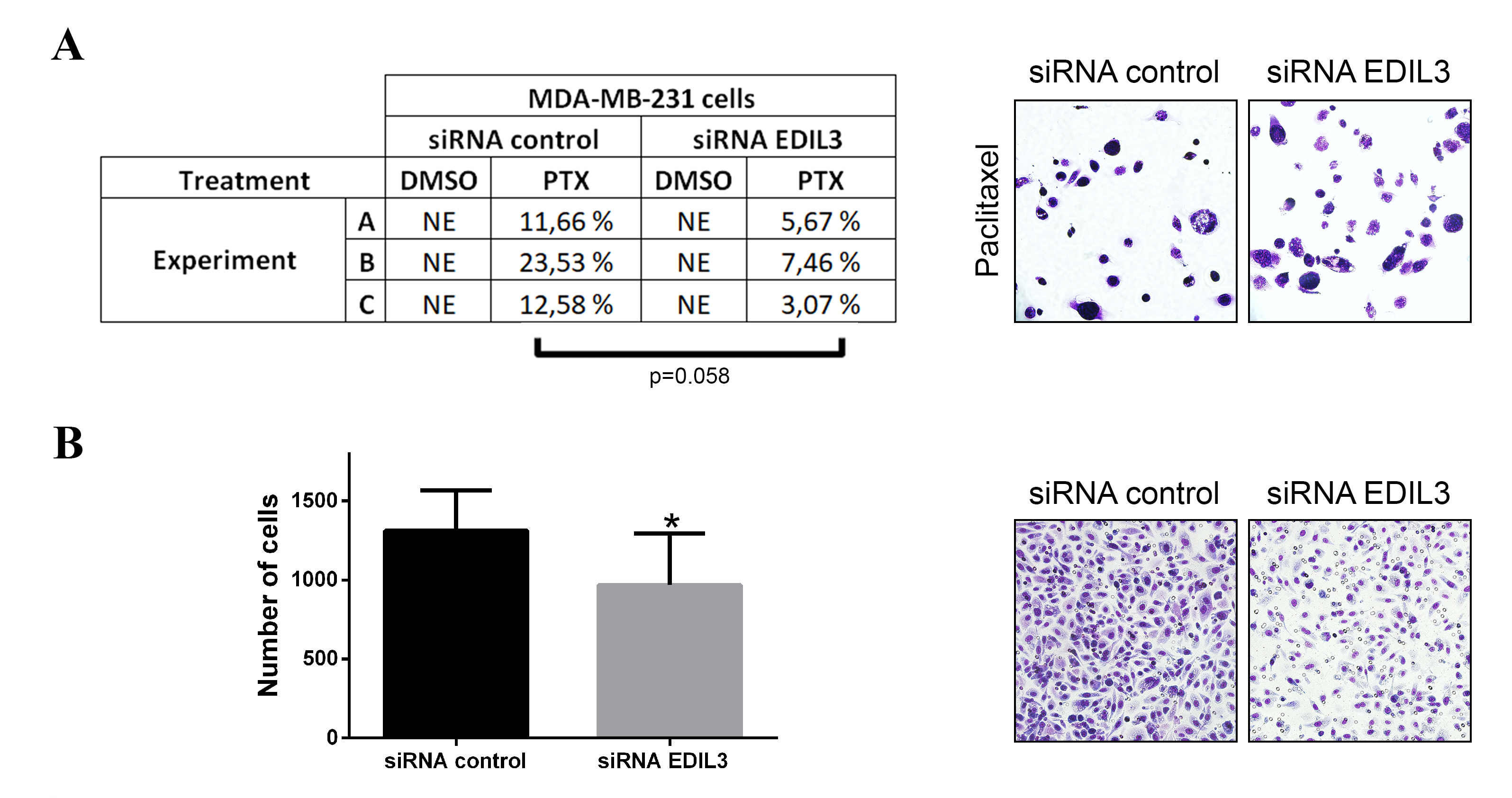

Supplement: Supplementary file 3 — Supplementary Figure S3-20-0879R [file 41420_2020_322_MOESM3_ESM.tif]

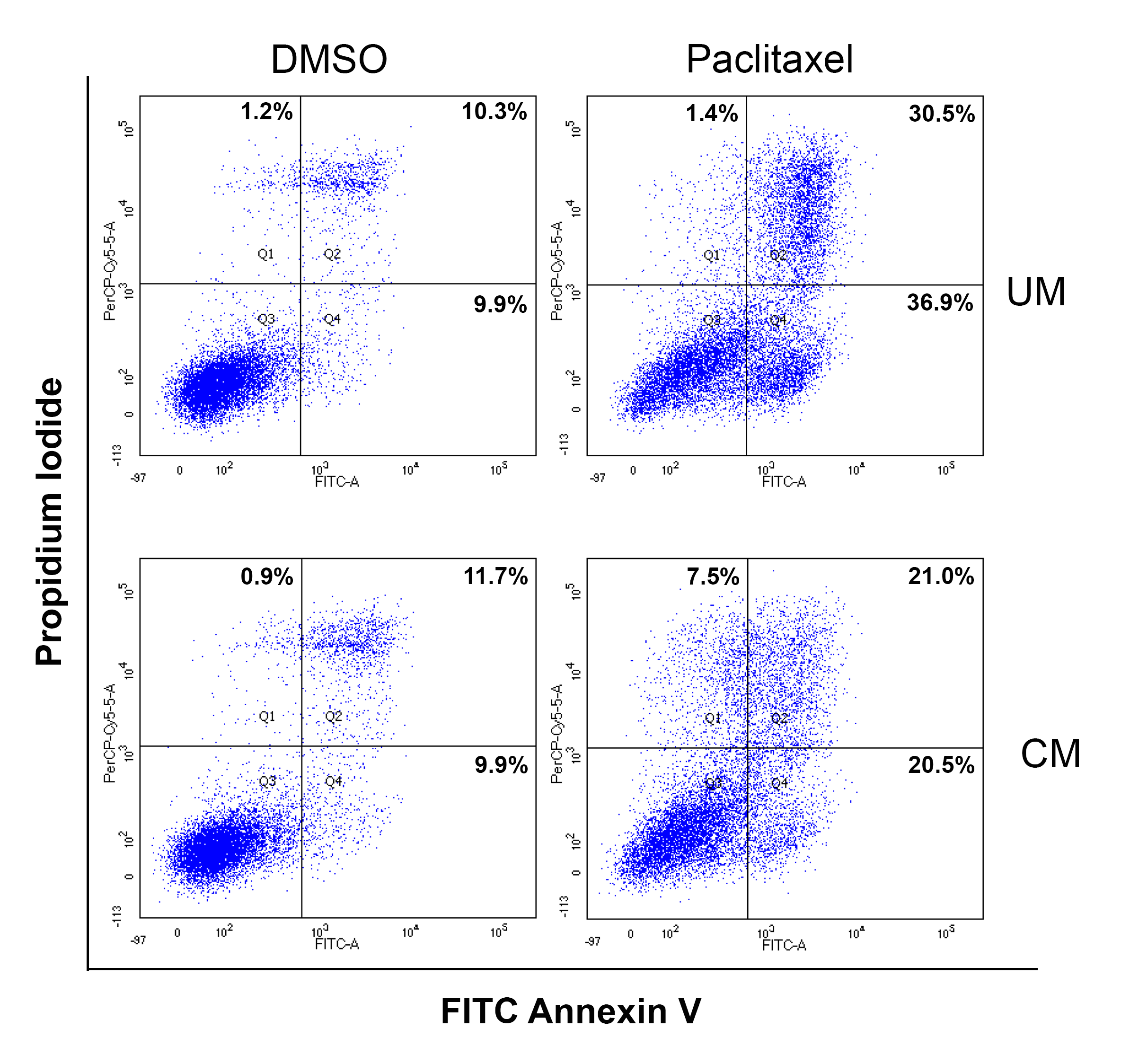

Supplement: Supplementary file 4 — Supplementary Figure S4-20-0879R [file 41420_2020_322_MOESM4_ESM.tif]

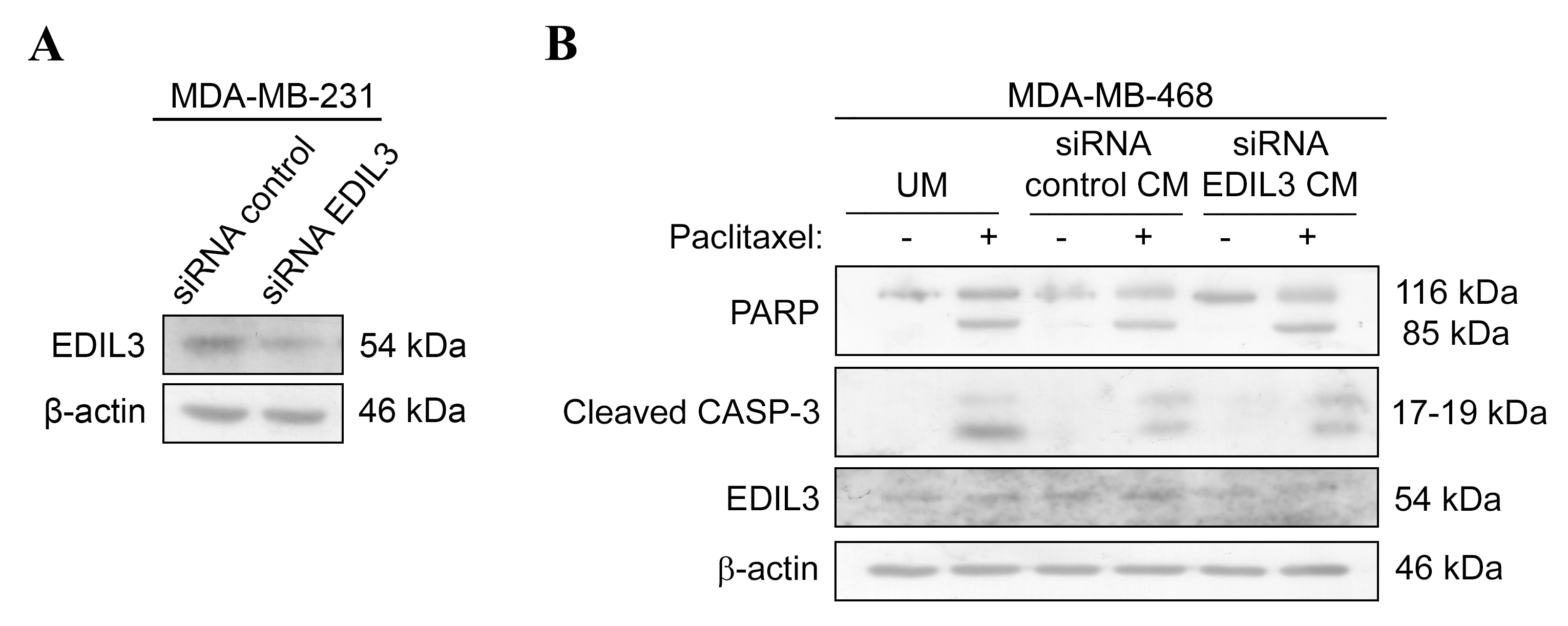

Supplement: Supplementary file 5 — Supplementary Figure S5-20-0879R [file 41420_2020_322_MOESM5_ESM.tif]

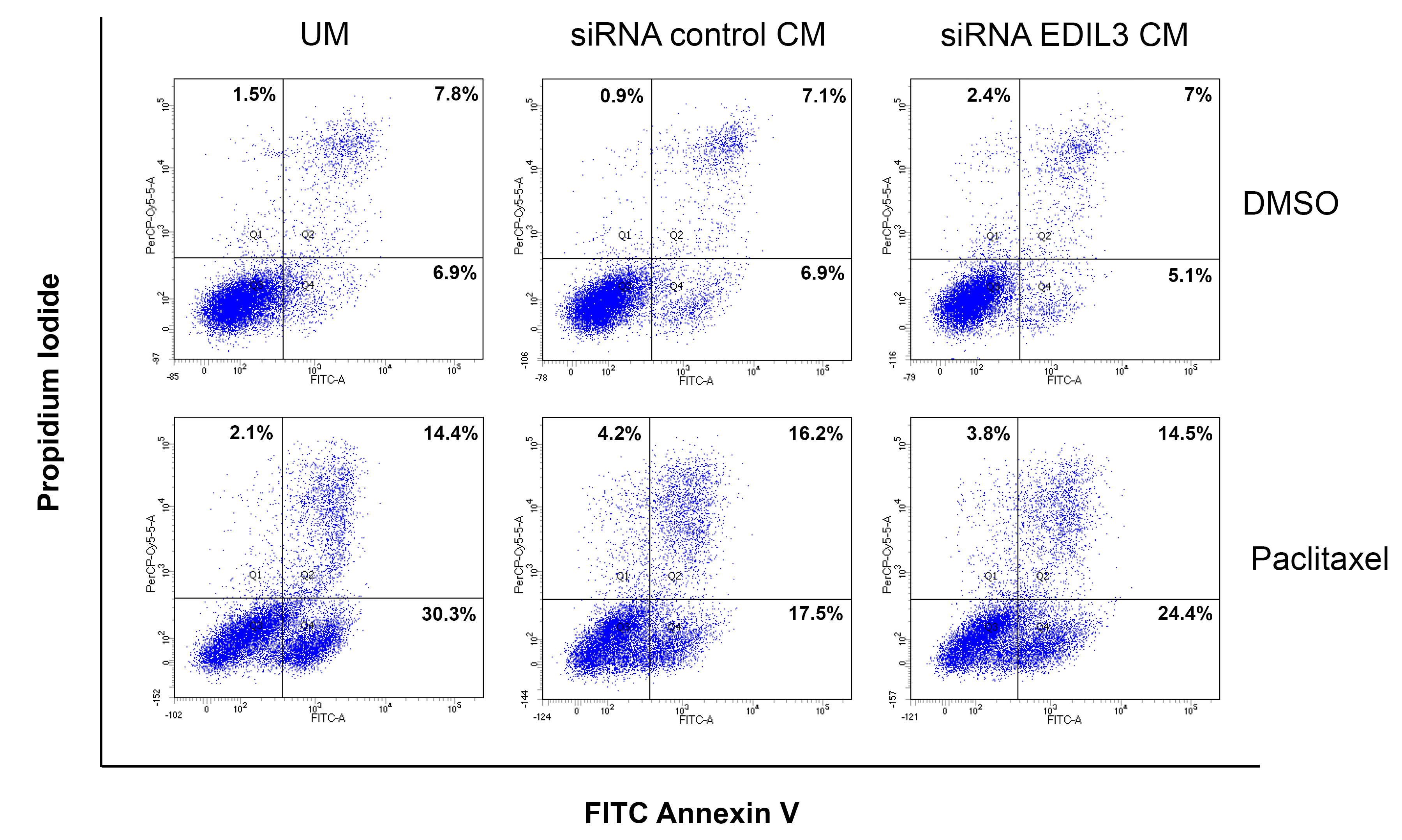

Supplement: Supplementary file 6 — Supplementary Figure S6-20-0879R [file 41420_2020_322_MOESM6_ESM.tif]

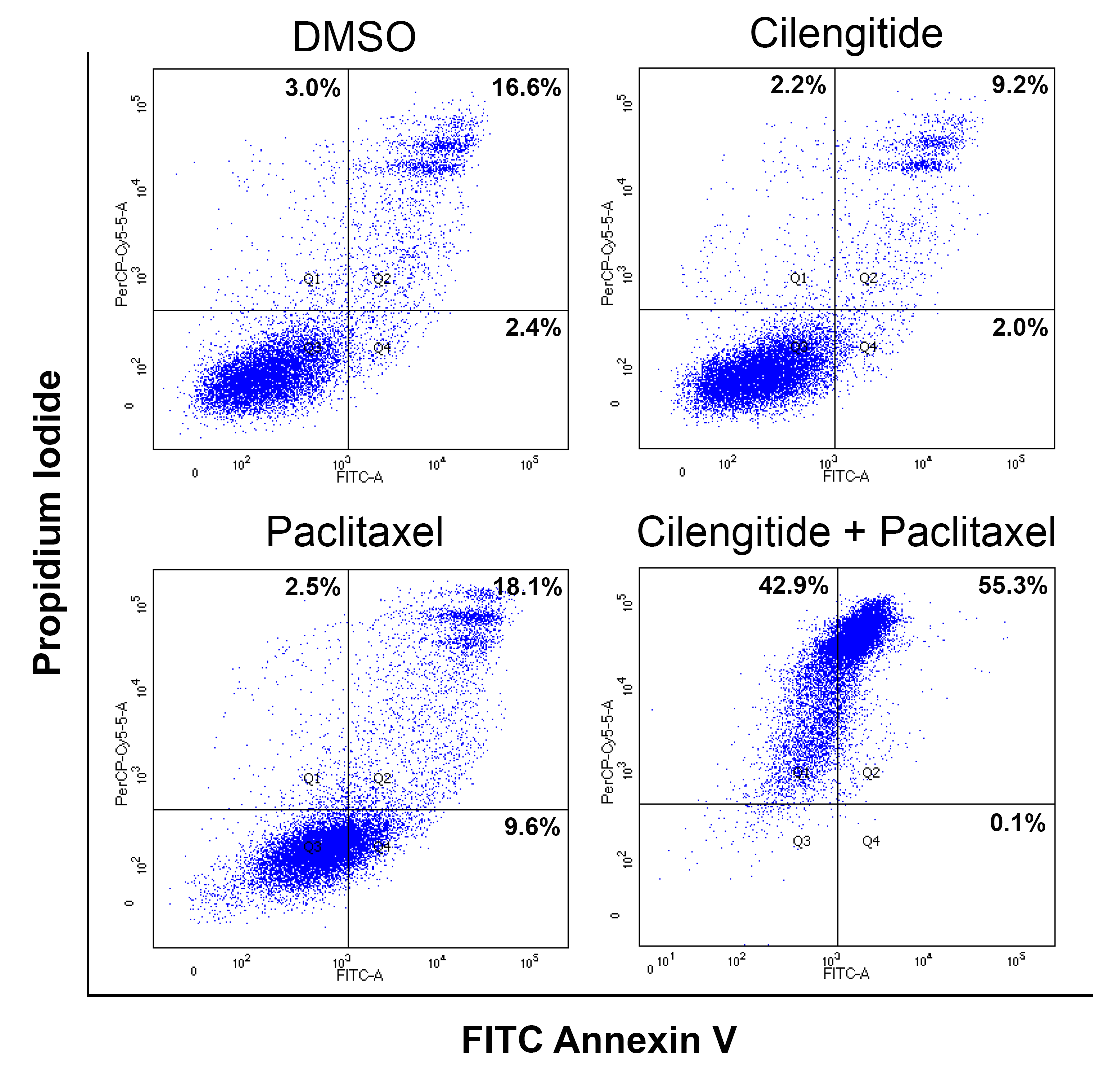

Supplement: Supplementary file 7 — Supplementary Figure S7-20-0879R [file 41420_2020_322_MOESM7_ESM.tif]
